# Pattern and Appropriateness of Antimicrobial Prescriptions for Upper Respiratory Tract and Dental Infections in Male Prisoners in Italy

**DOI:** 10.3390/antibiotics10111419

**Published:** 2021-11-20

**Authors:** Gabriella Di Giuseppe, Raffaele Lanzano, Armando Silvestro, Francesco Napolitano, Maria Pavia

**Affiliations:** Department of Experimental Medicine, University of Campania “Luigi Vanvitelli”, 80138 Naples, Italy; gabriella.digiuseppe@unicampania.it (G.D.G.); raffaele.lanzano.md@gmail.com (R.L.); armando.silvestro@studenti.unicampania.it (A.S.); francesco.napolitano2@unicampania.it (F.N.)

**Keywords:** antibiotic prescribing, antimicrobial resistance, prisoners, upper respiratory tract infections, dental infections

## Abstract

Background: This study explored the antimicrobial prescribing pattern for upper respiratory tract and dental infections in prisoners in Italy, with specific attention paid to the appropriateness of indication and its potential determinants. Methods: This investigation was conducted through the consultation of clinical records of adult male inmates in a prison in the south of Italy. Results: Prescription of antimicrobials for upper respiratory tract infections ranged from 41.9% in influenza diagnoses to 88% in pharyngitis diagnoses, with high prevalence also for bronchitis (73.5%) and common cold (57.7%), and those for dental infections ranged from 82% in pulp necrosis and symptomatic apical periodontitis/pulp necrosis and localized acute apical abscess diagnoses, to 85.7% in symptomatic irreversible pulpitis with or without symptomatic apical periodontitis diagnoses. The most frequently prescribed antimicrobial was amoxicillin and clavulanic acid (33.8%), followed by amoxicillin (26.5%), macrolides (19.8%) and third-generation cephalosporins (7.9%). The overall antimicrobial overprescription was 69.4%, whereas an antimicrobial prescription was provided in all 52 cases in which it was indicated. The inappropriate antimicrobial prescriptions were significantly less likely for bronchitis, influenza and symptomatic irreversible pulpitis with or without symptomatic apical periodontitis compared to common cold/pharyngitis/rhinosinusitis, and when the antimicrobial prescription was provided by medical specialists compared to prison physicians, whereas antimicrobial overprescriptions without indications were significantly more frequent in patients with underlying chronic clinical conditions. Conclusions: A concerning widespread practice of inappropriate antimicrobial prescriptions in prisoners was found. Diagnoses-specific monitoring of antimicrobial use and prison-focused antimicrobial stewardship policies are strongly needed.

## 1. Introduction

Since their discovery and introduction in the clinical practice, antimicrobials have demonstrated their extraordinary therapeutic effectiveness, as well as their potential for the selection of resistant microorganisms. It has been demonstrated that the use of antimicrobials, whether “appropriate” or “inappropriate,” can contribute to the development of antimicrobial resistance (AMR), which has been classified by WHO in the list of the ten top threats to global health [1]. In Italy, the National Report on Antibiotics Use in Italy, issued by The Medicines Utilisation Monitoring Centre of the Italian Medicines Agency, has reported that in 2019, antimicrobials were the most-used group of drugs in the population, with 4 out of 10 subjects having received an antimicrobial prescription, mainly prescribed by primary care physicians, accounting for 3.6% of the total drugs expenditure and 1.5% of the total drug consumption within the National Health Service (NHS) [2]. Although AMR is alarming for the whole population, it is likely to pose more serious threats to groups at greater overall risk of contracting infections or infectious diseases, and indeed appropriateness of antimicrobial prescribing for therapeutic or prophylactic purposes has been thoroughly investigated in more vulnerable populations, such as hospitalized patients [3], subjects with underlying clinical conditions, namely chronic conditions such as heart disease, cancer, hypertension, diabetes, etc. [4,5], institutionalized elderly [6,7], and children [8].

Due to prisons and prisoners’ characteristics, such as overcrowding, compromised hygiene conditions, attendance of poorly ventilated areas, reduced access to diagnostic tests and healthcare services, as well as their compromised health status compared with the general population [9,10], incarcerated subjects are more at risk of developing infectious diseases, specifically those spread by airborne transmission, and, as a consequence, to the widespread use of antimicrobials. However, the antimicrobial prescribing pattern in prisoners is almost unexplored [11]. This is concerning, since it has been reported that among the most frequently diagnosed diseases in the detained populations in Italy, there are respiratory tract infections (RTI), mainly acute upper RTI (URTI) and oral diseases [12], which are among the conditions at high risk of antimicrobial overprescription. Specifically, acute URTI were the second leading cause of morbidity (24.4%) in detained subjects, and the most frequently used drugs for these conditions were systemic antimicrobials (23%). Moreover, oral diseases were the first leading cause of morbidity among gastrointestinal diseases (39.7%), and in this case antimicrobials were among the most used drugs too [12].

To combat the overprescription and inappropriate use of antimicrobials, a series of recommendations targeted to URTI [13,14,15], and dental infections [16] have been issued, and guidelines specifically addressed to antimicrobial stewardship in prisons were published in the USA in 2013, then updated in 2019 [17]. All these guidelines recommend a wise use of antimicrobials, with the aims of improving patient outcomes, decreasing unnecessary antimicrobial use, counteracting the development of AMR, and decreasing unintentional antimicrobial adverse effects.

To fill this knowledge gap, this study was aimed at exploring the antimicrobial prescribing pattern for URTIs and dental infections in prisoners in Italy, with specific attention paid to the appropriateness of indication and its potential determinants.

## 2. Results

Out of the 971 selected clinical records of the 2046 prisoners that were incarcerated at the time of the study, 311 (32%) reported one or more of the diagnoses of interest. In particular, in 150 records prisoners had one of the selected diagnoses, in 71 two, in 49 three, in 21 four, and in 20 five or more of the selected diagnoses were retrieved, for a total of 637 diagnoses.

The selected 311 prisoners had a mean age of 41.8 years (Standard Deviation (SD) ± 11.4, range 19–76), almost all (98.1%) were Italian, more than half (56.1%) had been detained for two years or less, almost half (49.2%) were affected by underlying chronic clinical conditions, and 23.1% took chronic medications; moreover, allergies to antimicrobials were reported for 2.6% and to other drugs for 2.5% detainees (Table 1).

Of the total 637 retrieved diagnoses, 368 (57.8%) were URTI and 269 (42.2%) dental infections. Among the URTI, 142 (22.3%) were pharyngitis, 136 (21.3%) acute bronchitis, 62 (9.7%) influenza, 26 (4.1%) common cold, and 2 (0.3%) were rhinosinusitis, whereas among dental infections 147 (23.1%) were symptomatic irreversible pulpitis with or without symptomatic apical periodontitis, and 122 (19.2%) were pulp necrosis and symptomatic apical periodontitis/pulp necrosis and localized acute apical abscess. The most-often coexisting diagnoses that occurred in prisoners were symptomatic irreversible pulpitis and pulp necrosis and localized acute apical abscess (12.5%), pharyngitis and acute bronchitis (11.6%), and pharyngitis and pulp necrosis and localized acute apical abscess (9%).

In the vast majority of cases (83%), diagnoses and prescriptions were performed by prison physicians, whereas the remaining were provided by specialists, such as dentists and pneumologists. Diagnoses were mostly based on clinical signs and symptoms, and only in eight (1.3%) cases a chest X-ray was performed, in six (1%) an orthopantomography, and in two (0.3%) a rapid streptococcal test (RST), whereas no throat swab culture prescription was found in the clinical records. Moreover, for 161 (25.4%) diagnoses there had been one or more consultations by a physician in the previous four weeks.

Table 2 shows the pattern of antimicrobials prescribed overall and according to the different diagnoses. In 494 (77.5%) diagnoses an antimicrobial was prescribed, specifically in 268 (72.8%) URTI and in 226 (84%) dental infections. The mean duration of antimicrobial therapy was 5.4 days (SD ± 1, range 1–10), the route of administration was oral in 84.8% and intramuscular in the remaining 15.2% cases, and in 35.3% of diagnoses an antimicrobial therapy had been practiced in the previous four weeks. Moreover, in 147 (23.1%) cases an anti-inflammatory drug was prescribed, in 48 (7.5%) a mucolytic drug was prescribed, and in 44 (6.9%) a dental treatment was performed. 

Prescriptions of antimicrobials for URTI ranged from 41.9% of influenza diagnoses to 88% of pharyngitis diagnoses, with high prevalence also for bronchitis (73.5%) and common cold (57.7%), and those for dental infections from 82% of pulp necrosis and symptomatic apical periodontitis/pulp necrosis and localized acute apical abscess diagnoses to 85.7% of symptomatic irreversible pulpitis with or without symptomatic apical periodontitis diagnoses. The most frequently prescribed antimicrobial was amoxicillin and clavulanic acid (33.8%), followed by amoxicillin (26.5%), macrolides (19.8%) and third-generation cephalosporins (7.9%). Amoxicillin and clavulanic acid were also the most frequently prescribed antimicrobials in symptomatic irreversible pulpitis with or without symptomatic apical periodontitis (40.5%), pharyngitis (40.8%), common cold (33.3%) and influenza (53.8%), whereas amoxicillin was the most chosen antimicrobial for pulp necrosis and symptomatic apical periodontitis/pulp necrosis and localized acute apical abscess (46%), and ceftriaxone was the most chosen for bronchitis (26%); moreover macrolides and fluoroquinolones accounted for 31% and 21% of antimicrobial prescriptions in bronchitis (Table 2).

**Figure 1:**
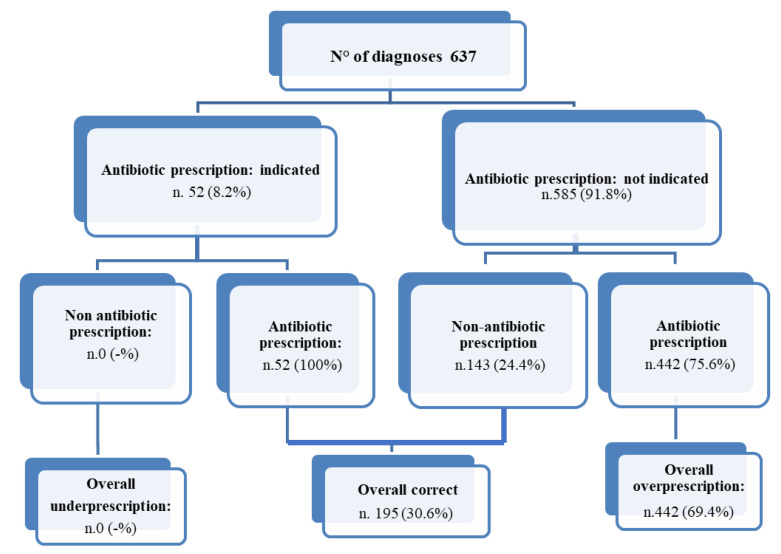
Appropriateness of antimicrobial prescriptions according to indication in the selected diagnoses.

Figure 1 displays the appropriateness of antimicrobial prescriptions according to indication. Of the total 494 antimicrobial prescriptions, 442 were not indicated, with an overall antimicrobial overprescription of 69.4%, whereas an antimicrobial prescription was provided in all 52 cases in which it was indicated, specifically in two cases of bronchitis and 50 cases of pulp necrosis and localized acute apical abscess. Therefore, an appropriate prescription pattern was recorded in only 195 (30.6%) of all diagnoses, and no underprescription was encountered. Regarding the specific diagnoses, an overprescription of antimicrobials was found in 88% of pharyngitis, 85.7% of symptomatic irreversible pulpitis with or without symptomatic apical periodontitis, 72.1% of bronchitis, 57.7% of common cold, 41.9% of influenza diagnoses, and 41% of pulp necrosis and symptomatic apical periodontitis/pulp necrosis and localized acute apical abscess, moreover the only two rhinosinusitis cases were inappropriately treated with antimicrobials. 

The analysis restricted to the 52 appropriate prescriptions according to indication showed that in 41 (78.8%) cases, the chosen antimicrobial and the duration of treatment was inappropriate, as well as the dose in 44 (88%) and the route of administration in 3 (5.8%) cases. The inappropriately chosen molecules prescribed in the 52 indicated therapies belonged to several antimicrobial classes, among which were aminopenicillins, macrolides, cephalosporins and fluoroquinolones.

Table 3 displays the results of the univariate and multivariate analyses investigating determinants of inappropriate antimicrobial prescriptions (overprescriptions) by indication. Several variables were associated with inappropriate antimicrobial prescriptions, including type of diagnosis, type of healthcare professional who made the prescription, occurrence of medical consultations and antimicrobial prescriptions in the previous four weeks, and presence of underlying chronic clinical conditions. Specifically, inappropriate antimicrobial prescriptions were significantly less likely for bronchitis (Odds Ratio (OR) = 0.28, 95% Confidence Interval (CI) = 0.13–0.61), influenza (OR = 0.09, 95% CI = 0.03–0.21) and symptomatic irreversible pulpitis with or without symptomatic apical periodontitis (OR = 0.02, 95% CI = 0.01–0.04) compared to common cold/pharyngitis/rhinosinusitis, and when the antimicrobial prescription was provided by medical specialists (OR = 0.25, 95% CI = 0.10–0.60) compared to prison physicians, whereas antimicrobial overprescriptions without indications were significantly more frequent in patients with underlying chronic clinical conditions (OR = 2.15, 95% CI = 1.12–4.12) and when a medical consultation (OR = 3.80, 95% CI = 1.80–8.03) or an antimicrobial prescription (OR = 6.56, 95% CI = 2.21–19.50) had occurred in the previous four weeks.

## 3. Discussion

This study has provided a comprehensive recognition and has produced novel knowledge on the appropriateness of antimicrobials use, focusing on detained subjects, which are a vulnerable, but virtually unexplored population regarding this concerning issue. The results of this investigation offer interesting suggestions on the determinants of inappropriate use, fostering decision making on the most effective interventions to be put in place to combat the development of AMR in the investigated context.

The selected diagnoses were chosen since they have been reported as among the most frequently occurring in prisoners, and due to their frequent viral origin, at a high risk of antimicrobial inappropriate use. According to the findings of this study, the overprescription of antimicrobials is widespread in this population, accounting for an estimated 69.4% of selected URTI and dental infections, which were treated with an antimicrobial without an indication. Comparisons with antimicrobial prescription practices in analogous contexts are challenging, since, to the best of our knowledge, antimicrobial use in detained subjects has been investigated only in one study, reporting antimicrobial prescriptions in 67% of URTI and in 3.2% of prisoners with influenza symptoms [11]. The extent of inappropriate use of antimicrobials is alarming, but to some extent expected, since a study conducted in the same area has documented a high frequency of antimicrobial overprescription for URTI in adult primary care (66.5%) [18]. It should also be noted that the comparison of these results with some of the disease-specific antibiotic prescribing quality indicators (APQI) proposed by the European Surveillance of Antimicrobial Consumption (ESAC) project to assess the quality of antimicrobial prescribing in primary care [19] highlights a very worrying scenario, since antimicrobial prescriptions for bronchitis are considered in an acceptable range when prescriptions of systemic antimicrobials and of quinolones do not exceed 30% and 5% of cases, respectively, whereas in the investigated prison they were prescribed in 73.5% and 21% of bronchitis diagnoses, respectively. Relevant deviations from the APQI have also been reported in a study conducted in primary care settings in Belgium, which investigated the adherence of antimicrobial prescriptions to all proposed APQI [20].

Of special concern is the extremely frequent use of broad-spectrum antimicrobials, which has been repeatedly discouraged by guidelines aimed at a wise use of antimicrobials [21,22]. It should be argued, however, that this practice is not specific to this setting, but is one of the most frequently reported problems in the literature investigating antimicrobial use; indeed, consumption of broad spectrum antimicrobials that are not recommended for routine use because of their high potential for development of resistance rose worldwide by 90.9% in the period 2000–2015 [23], and the suggested reasons include several factors, including poor antimicrobial stewardship, as well as the consequence of a rise in infections that are resistant to the narrower-spectrum first- and second-line antimicrobials. It should also be acknowledged, however, that in Italy, narrow-spectrum penicillin is not available, due to high frequency of AMR and consequential supply shortage. A similar situation has been reported in Belgium, where low use of narrow-spectrum penicillin was related to industry stock cuts [20].

Most of the diagnoses relied only on clinical signs and symptoms, and this pattern is in line with the other study performed in prisoners, who received only a clinical diagnosis in 88.7% of cases [11]. Use of diagnostic tests, and particularly of rapid point-of-care tests is a potential tool to counteract overuse and misuse of antimicrobials, although it has been reported that it should be coupled with antimicrobial stewardship [24,25]. The extremely low use of diagnostic and rapid point-of-care tests in this study is not surprising, it has been repeatedly reported in Italy, and the suggested causes were the associated costs, since they are not prescribed free of charge [18,26].

The finding that inappropriate antimicrobials were significantly more likely to be prescribed by prison’s physicians when compared to other professionals is probably expected, but nevertheless concerning, underlying the need of a more thorough assessment of reasons promoting this practice. Since data on diagnoses and prescriptions were extracted from clinical records, we could not investigate reasons for inappropriate prescriptions, and it would be interesting to assess whether this behaviour was the result of physicians’ poor knowledge on URTI and dental antimicrobial treatment guidelines, or to a more cautious approach induced by their concern for the peculiar prison context. Indeed, reasons for prescribing antimicrobials may be related to a perceived higher conceivable risk of development of more serious bacterial infections by prisoners, or to the potential requirement for increased follow-up when antimicrobials are not prescribed; moreover, it has been reported that not prescribing antimicrobials is perceived by patients as “not being treated”, coupled by the misconception that antimicrobials are harmless [27]. Further research investigating knowledge and attitudes of prison physicians regarding antimicrobial prescriptions, as well as on reasons for inappropriately prescribing antimicrobials in this context would be worthwhile. As already reported in studies conducted in the community [18,20,28,29], antimicrobial prescribing pattern and related appropriateness was associated with type of diagnoses, with pharyngitis, common cold and rhinosinusitis showing a significantly higher odds of being inappropriately treated with antimicrobials compared to bronchitis and influenza, as well as to symptomatic irreversible pulpitis with or without symptomatic apical periodontitis. It is well-known that these conditions rarely benefit of the use of antimicrobials, given their frequent viral origin and self-limiting nature, and this finding suggests the need for a more thoughtful attention to these diagnoses in the implementation of antimicrobial stewardship programs in prisons.

Another interesting determinant of inappropriate use of antimicrobials revealed by the study was the presence of an underlying chronic clinical condition. Patients with comorbidities are at high risk of developing AMR for their vulnerability to infection and related frequent exposure to antimicrobial treatments; therefore, an even more prudent use of these drugs should be warranted. However, the presence of comorbidities has also been found to be an independent driver of antimicrobial prescribing in a study by Shallcross et al., investigating the role of comorbidities in the decision to prescribe antimicrobials in primary care settings [30], where the authors conclude that there is a need to understand whether higher rates of antimicrobial use in patients with comorbidities are primarily driven by diagnostic uncertainty or by concerns about an overall increased susceptibility to infection in these patients. Similar results have been found in elderly subjects with comorbidities [31] and in Swedish primary care patients [32]. According to these results, since prisoners are a subpopulation at high risk of comorbidities [33], a specific attention to antimicrobial prescribing in this subset of prisoners should be given in antimicrobial stewardship programs oriented to rationalize antimicrobial use in prisoners. Indeed, a multidisciplinary antimicrobial stewardship program implemented in US prisons has been demonstrated to be effective, contributing to a decrease in both the total number and the rate of antimicrobial prescriptions from 2010 to 2015 [27].

All taken together, the findings of this study have contributed to underlining the strategic role of prisoners as a unique reservoir for the development and spread of AMR. Indeed, the peculiar conditions of confinement as well as their overall poor health status expose prisoners to (1) high frequency of infections and infectious diseases, (2) large use of often inappropriate antimicrobials, (3) large spread of resistant microorganisms within the inmates, and (4) due to the rapid turnover of the prisons, widespread diffusion of resistant microorganisms into the community.

### Strengths and Limitations of the Study

Many investigations have explored the frequency of use of antimicrobials in many settings and for many specific conditions [34,35] but very few could evaluate the appropriateness of individual prescriptions according to indication, and in no cases among detained subjects, and these two peculiarities represent a relevant strength of this investigation. The need to have access to antimicrobial consumption data that are related to clinical information has been recently emphasized in a study investigating the quality of antimicrobial consumption in the community in the European countries, as a relevant requisite to better understand prescribing habits and to identify opportunities for improvement [36].

However, the interpretation of results should also take into account potential limitations. First of all, data were retrieved from clinical records, and the indication of antimicrobial prescriptions relied on the completeness of the reported data. Therefore, it cannot be excluded that an overestimation of the inappropriate use of antimicrobials might have resulted from the omission of information on diagnostic tests or clinical manifestations justifying the antimicrobial prescriptions; nevertheless, the extent of inappropriate use of antimicrobials was so high that it could not be substantially modified by sparse incomplete information in the clinical records. Moreover, it should be acknowledged that the investigation involved only one prison in southern Italy, therefore caution on generalizability of results is plausible; however, prison physicians, who were the predominant healthcare professionals involved in prisoners’ care, serve many prisons in the area, therefore we believe there would not be relevant differences at least in the prisons of southern Italy. Additionally, it may be argued that the extensively reported inappropriateness of prescribed antimicrobials may be the consequence of the behaviour of just a few physicians involved in healthcare of this specific prison. However, in Italy, physicians serving prisons are the same that work within the NHS; therefore, there is a substantial turnover, with many different physicians providing prisoners’ healthcare. Moreover, data on antimicrobial prescriptions refer to a wide time period, therefore we do not believe that the inappropriateness of prescriptions may be only related to just one or few prescribers. Furthermore, the study was carried out in a prison that hosted only males, whereas a meta-analysis of the literature has reported that use of antimicrobials in the community is more frequent in females, especially for RTI, although the author concluded that there is no sufficient evidence in the gender epidemiology of infectious diseases that can explain the substantial difference they found [37]. Therefore, an even higher antimicrobial use could have been found if incarcerated women would have also been investigated. Finally, since clinical records of prisoners detained at the time of the study were analysed for the diagnoses and related prescriptions given in the previous three years, prisoners who had a longer stay may have contributed differently to those with a shorter incarceration. Nevertheless, since the duration of detention was not a determinant of inappropriate antimicrobial prescribing, the methods of diagnoses selection do not appear to have distorted the results.

## 4. Materials and Methods

### 4.1. Setting

This investigation was conducted between March 2021 and June 2021 through the consultation of clinical records of adult male inmates in the largest prison in the geographic area of Campania region, in the south of Italy, which in the study period hosted 2046 prisoners. According to the Italian legislation [38] every prisoner at the time of incarceration undergoes a medical consultation, and a clinical record is compiled and then updated for every healthcare need during incarceration (medical consultations, diagnostic tests, prescriptions, etc.). Physicians working within the NHS and specifically dedicated to inmates’ healthcare provide primary care and may refer patients to specialists or hospitals whenever needed.

### 4.2. Study Design and Data Collection

This study is part of a larger project developed by the University of Campania “Luigi Vanvitelli” and the Joint Operational Unit for “Health Protection at Prison Institutions”, to investigate several health-related issues in the prison population [39]. The study protocol was submitted to the director of the prison to obtain the access to prisoners’ clinical records, and complete anonymity and confidentiality of inmates’ data were guaranteed. Once the approval had been obtained, 7 out of the 12 prison pavilions were randomly selected, and all the clinical records of the inmates hosted in the selected pavilions (1100) were studied. Among these, only the clinical records reporting one or more of the most common upper respiratory tract (acute rhinosinusitis, pharyngitis, bronchitis, common cold, and influenza) and dental (symptomatic irreversible pulpitis with or without symptomatic apical periodontitis and pulp necrosis and symptomatic apical periodontitis/pulp necrosis and localized acute apical abscess) infections occurring between March 2018 and June 2021 were retrieved. The selected clinical records were reviewed by two investigators who were not directly involved in inmates’ care, and were summarized on a structured data extraction form.

### 4.3. Data Collection Instrument

The structured data extraction form was developed to collect the following information from the selected clinical records: (1) prisoners’ demographic, anamnestic and detention characteristics, such as age, nationality, months spent in detention, presence and type of underlying chronic clinical conditions, previous antimicrobial and/or other drug allergies, antimicrobial prescriptions in the previous four weeks, and current therapy(ies); (2) data on the diagnoses: type, characteristics of the healthcare professional who made the consultation (prison physician, specialist, emergency room physician, etc.), and diagnostic tests performed (throat swab culture, RST, C-reactive protein, X-ray, etc.); (3) information about antimicrobial prescriptions (type of antimicrobial, length of the therapy, dose and route of administration); (4) other medical consultations and prescriptions in the previous four weeks.

### 4.4. Outcome

Appropriateness of antimicrobial prescription patterns was assessed according to international guidelines [15,16,17]. In particular, antimicrobial treatment is indicated: (1) for acute rhinosinusitis, in presence of severe (>3–4 days) symptoms (fever ≥ 39 °C/102 °F and purulent nasal discharge or facial pain); persistent (>10 days) symptoms (nasal discharge or daytime cough) without improvement, or worsening (3–4 days) symptoms (worsening or new onset fever, daytime cough, or nasal discharge after initial improvement of a viral upper respiratory infections lasting 5–6 days); (2) for pharyngitis, only in presence of Group A beta-hemolytic streptococcal (GAS) infection; (3) for acute bronchitis, only for patients with acute bacterial exacerbation of chronic bronchitis and Chronic Obstructive Pulmonary Disease (COPD); (4) for common cold, and influenza, antimicrobial treatment is never indicated; (5) for symptomatic irreversible pulpitis with or without symptomatic apical periodontitis, antimicrobial treatment is never indicated; (6) pulp necrosis and symptomatic apical periodontitis/pulp necrosis and localized acute apical abscess, only if the symptoms worsen pending Definitive Conservative Dental Treatment (DCDT). Inappropriateness by indication was evaluated to assess both over- and under-prescription, and overprescription was defined as a prescription of an antimicrobial without indication and underprescription as no antimicrobial prescription in presence of an indication. Therefore, the prescription pattern was considered appropriate when an antimicrobial was prescribed when indicated and not prescribed when not indicated. Moreover, when an antimicrobial prescription was indicated, the appropriateness of the chosen molecule, length of the therapy and route of administration were also evaluated according to the international guidelines [13,15,16,17].

### 4.5. Pilot Study and Ethical Statement

The data collection instrument was pretested on a random sample of 50 clinical records included in the final sample, and the necessary changes were made before starting the study. In particular, duration of underlying chronic clinical conditions and previous accesses to healthcare facilities were eliminated from the final data collection instrument. Ethical approval was obtained by the Ethics Committee “Campania Centro” of the Local Health Unit Napoli 1 (protocol code: 297).

### 4.6. Statistical Analysis

Statistical analyses were carried out using Stata version 15 software [40]. Following a descriptive analysis of the study data to describe the sociodemographic and anamnestic characteristics of the prisoners, univariate analysis was performed using a chi-squared test and Student’s t-test for categorical and continuous variables, respectively. Normality has been assessed using the Shapiro–Wilk test. Then, multilevel mixed-effects logistic regression analysis was performed to investigate the independent characteristics associated with the inappropriate antimicrobial prescribing pattern by indication for the selected diagnoses (no = 0; yes = 1). The following independent variables were included in the model: age, in years (≤30 = 1; 31–50 = 2; >50 = 3), number of months spent in detention (≤12 = 1; 13–24 = 2; >24 = 3), presence of underlying chronic clinical conditions (0 = 0; ≥1 = 1), medications for underlying chronic clinical conditions (no = 0; yes = 1), type of diagnosis (common cold/pharyngitis/rhinosinusitis = 1; bronchitis = 2; influenza = 3; symptomatic irreversible pulpitis with or without symptomatic apical periodontitis = 4; pulp necrosis and symptomatic apical periodontitis/pulp necrosis and localized acute apical abscess = 5), physicians who prescribed antimicrobial therapy (prison physician = 0; medical specialists = 1), having had a medical consultation in the previous four weeks (no = 0; yes = 1), and having had an antimicrobial prescription in the previous four weeks (no = 0; yes = 1). With the aim of accounting for the multilevel dataset structure (diagnoses were “nested” within prisoners), the variable prisoner was introduced in the model as random factor.

All inferential tests were performed through the execution of bilateral hypothesis test with statistical significance level of *p* values equal to or less than 0.05. The results of univariate and multilevel mixed-effects logistic regression analyses were reported as ORs (crude and adjusted) and 95% Cis.

## 5. Conclusions

The findings of this study have revealed a concerning widespread practice of inappropriate antimicrobial prescriptions in prisoners, and has discovered potential determinants that should be the focus of further research. There is a need for diagnoses-specific monitoring of antimicrobial use coupled with evidence-based prison-focused antimicrobial stewardship policies to contrast the potential development of AMR in prisons.

## Figures and Tables

**Table 1:** Sociodemographic and anamnestic characteristics of the study population (N = 311).

Characteristics		
*Sociodemographics*	N	%
**Age, years**	41.8 ± 11.4 (19–76) *	
<31	59	19.0
31–50	184	59.1
>50	68	21.9
**Nationality (307) ^a^**		
Italians	301	98.1
Foreigners	6	1.9
**Length of detention in the prison, months (305) ^a^**	25.4 ± 19.3 (1–94) *	
≤12	98	32.2
13–24	73	23.9
>24	134	43.9
***Anamnestic***		
**Underlying chronic clinical conditions (309) ^a^**		
Yes	152	49.2
No	157	50.8
**Chronic medications**		
Yes	72	23.1
No	239	76.9
**Allergies to antimicrobials**		
Yes	7	2.2
No	304	97.8
**Allergies to other drugs**		
Yes	8	2.6
No	303	97.4

^a^ In brackets are the number of available data for each item. * Mean ± Standard Deviation (Range).

**Table 2:** Antimicrobial prescriptions according to different diagnoses.

			Upper Respiratory Tract Infections (URTI)										Dental Infections			
Type of Prescribed Antimicrobial	Total n. 637		Pharyngitis n. 142 (22.3%)		Bronchitis n. 136 (21.3%)		Influenza n. 62 (9.7%)		Common Cold n. 26 (4.1%)		Sinusitis n. 2 (0.3%)		Symptomatic Irreversible Pulpitis with or Without Symptomatic Apical Periodontitis n. 147 (23.1%)		Pulp Necrosis and Symptomatic Apical Periodontitis/Pulp Necrosis and Localized Acute Apical Abscess n. 122 (19.2%)	
	N	%	N	%	N	%	N	%	N	%	N	%	N	%	N	%
Episodes with antibiotic prescribing	494	77.5	125	88.0	100	73.5	26	41.9	15	57.7	2	100	126	85.7	100	82.0
*Aminopenicillin*	298	60.3	87	65.6	22	22.0	20	76.9	6	40.0	1	50.0	94	74.6	73	73.0
Amoxicillin	131	26.5	31	24.8	4	4.0	6	23.1	1	6.7	-	-	43	34.1	46	46.0
Amoxicillin + clavulanic acid	167	33.8	51	40.8	18	18.0	14	53.8	5	33.3	1	50.0	51	40.5	27	27.0
*Macrolides*	98	19.8	33	26.4	31	31.0	6	23.1	7	46.6	1	50.0	17	13.5	3	3.0
Rovamycin	17	3.4	2	1.6	1	1.0	-	-	-	-	-	-	14	11.1	-	-
Clarithromycin	54	10.9	24	19.2	20	20.0	4	15.4	3	20.0	-	-	2	1.6	1	1.0
Azithromycin	27	5.5	7	5.6	10	10.0	2	7.7	4	26.6	1	50.0	1	0.8	2	2.0
*Cephalosporins*	39	7.9	3	2.4	26	26.0	-	-	1	6.7	-	-	3	2.4	6	6.0
Ceftriaxone	39	7.9	3	2.4	26	26.0	-	-	1	6.7	-	-	3	2.4	6	6.0
*Fluoroquinolones*	30	6.1	4	3.2	21	21.0	-	-	1	6.7	-	-	2	1.6	2	2.0
Levofloxacin	7	1.4	2	1.6	4	4.0	-	-	-	-	-	-	-	-	1	1.0
Ciprofloxacin	23	4.7	2	1.6	17	17.0	-	-	1	6.7	-	-	2	1.6	1	1.0
*Lincosamides*	29	5.9	3	2.4	-	-	-	-	-	-	-	-	10	7.9	16	16.0
Lincomycin	28	5.7	3	2.4	-	-	-	-	-	-	-	-	10	7.9	15	15.0
Clindamycin	1	0.2	-	-	-	-	-	-	-	-	-	-	-	-	1	1.0
Episodes with no antibiotic prescribing	143	22.5	17	12.0	36	26.5	36	58.1	11	42.3	-	-	21	14.3	22	18.0

**Table 3:** Results of univariate and multilevel mixed-effects logistic regression analysis exploring the characteristics associated with inappropriate antimicrobial prescribing pattern for the selected diagnoses.

Variable	Inappropriate Antimicrobial Prescribing		Univariate Analysis	Multivariate Analysis
	N	%	Crude OR (95% CI)	Adjusted OR (95% CI)
Type of diagnosis				
Common cold/pharyngitis/rhinosinusitis	142	83.5	1 *	1 *
Symptomatic irreversible pulpitis with or without symptomatic apical periodontitis	126	85.7	0.08 (0.05–0.17)	0.02 (0.01–0.04)
Influenza	26	41.9	0.09 (0.04–0.21)	0.09 (0.03–0.21)
Bronchitis	98	72.1	0.37 (0.18–0.75)	0.28 (0.13–0.61)
Pulp necrosis and symptomatic apical periodontitis/pulp necrosis and localized acute apical abscess	50	41	1.21 (0.57–2.53)	0.79 (0.35–1.76)
Having had a medical consultation in the previous four weeks				
No	310	65.7	1 *	1 *
Yes	131	81.4	2.75 (1.63–4.65)	3.80 (1.80–8.03)
Having had an antimicrobial prescription in the previous four weeks				
No	372	66.8	1 *	1 *
Yes	70	87.6	3.37 (1.59–7.16)	6.56 (2.21–19.50)
Physicians who prescribed antimicrobial therapy				
Prison physicians	370	66.7	1 *	1 *
Medical specialists	72	69.9	1.25 (0.75–2.11)	0.25 (0.10–0.60)
Presence of underlying chronic clinical conditions				
No	223	66	1 *	1 *
Yes	216	73	1.61 (1.01–2.57)	2.15 (1.12–4.12)
Number of months spent in detention				
≤12	119	75.8	1 *	1 *
13–24	92	64.8	0.54 (0.28–1.03)	0.49 (0.22–1.11)
>24	224	67.9	0.68 (0.39–1.18)	0.87 (0.42–1.77)
Age, years				
≤30	82	70.1	1 *	1 *
31–50	262	68.9	0.89 (0.48–1.65)	0.74 (0.34–1.63)
>50	98	70	1.10 (0.53–2.29)	0.72 (0.27–1.91)
Taking medications for underlying chronic clinical conditions				
No	330	69.5	1 *	1 *
Yes	112	69.1	1.10 (0.64–1.88)	1.01 (0.48–2.12)

* Reference category.

## Data Availability

The data presented in this study are available on request from the corresponding author.
